# Identification of key genes in periodontitis

**DOI:** 10.3389/fgene.2025.1579848

**Published:** 2025-03-19

**Authors:** Xianyang Cheng, Shan Shen

**Affiliations:** ^1^ School of Stomatology, Jinan University, Guangzhou, China; ^2^ Department of Stomatology, The First Affiliated Hospital of Jinan University, Guangzhou, China

**Keywords:** periodontitis, random forest, WGCNA, key genes, enrichment analysis

## Abstract

Periodontitis, a prevalent global oral health issue, is primarily characterized by chronic inflammation resulting from bacterial infection. Periodontitis primarily affects the tissues surrounding and supporting the teeth, encompassing the gingival tissue, periodontal attachment apparatus, and the bony socket. The disease mechanism results from intricate interactions between hereditary factors, the body’s defense mechanisms, and shifts in the composition of oral microbiota, with each element playing a crucial role in the initiation and advancement of the pathological process. The early symptoms of periodontitis are often not obvious, resulting in patients often not seeking medical attention until they are seriously ill, so finding biomarkers for periodontitis is essential for timely diagnosis and treatment. In this study, we selected two datasets (GSE10334 and GSE16134) by in-depth analysis of publicly available sequencing data of affected and unaffected gum tissue in periodontitis patients in the GEO database. To identify key genes associated with periodontitis pathogenesis and explore potential therapeutic biomarkers, we employed two complementary computational approaches: Random Forest, a robust machine learning algorithm for feature selection, and Weighted Gene Co-expression Network Analysis (WGCNA), a systems biology method for identifying co-expressed gene modules. Through comprehensive analysis of these combined datasets, our objective is to elucidate the underlying molecular pathways governing periodontal disease progression, thereby identifying novel therapeutic targets that may facilitate the design of improved clinical interventions for this condition. This study establishes a substantial scientific foundation that contributes to both clinical applications and fundamental research in periodontitis. The findings not only offer valuable insights for developing early diagnostic strategies and therapeutic interventions but also provide a robust theoretical framework to guide future investigations into the molecular mechanisms underlying this complex disease.

## 1 Introduction

Periodontal inflammation, a chronic condition triggered by microbial activity, is characterized by the progressive breakdown of the periodontal support system, including the gums, periodontal ligament, and alveolar bone, ultimately leading to compromised tooth stability and functionality (Manresa et al., 2018). Periodontitis affects people of all ages and is particularly common in adults. Epidemiological evidence demonstrates a significant positive correlation between periodontitis prevalence and advancing age, with disease incidence showing a progressive upward trend across successive age cohorts (Holde et al., 2017; Pischon et al., 2008; Hewlett et al., 2022; Eke et al., 2016). Global epidemiological data from 2021 revealed that severe periodontitis affected over 1 billion individuals worldwide, corresponding to an age-standardized prevalence rate of 12.50%. Projections indicate a substantial 44.32% increase in disease burden, with the affected population expected to exceed 1.5 billion cases by 2050, highlighting the growing global challenge of this oral health condition (Nascimento et al., 2024). According to statistics, the proportion of men suffering from periodontitis is usually higher than that of women, which may be associated with various contributing elements, including tobacco use patterns among males, dental care maintenance practices, and fluctuations in endocrine system regulation (Del Pinto et al., 2024; Kim et al., 2014; Michelson et al., 2022; Liu Y. et al., 2018). The development and progression of periodontal disease result from intricate interactions among diverse causative elements, with genetic predisposition, dysregulated host immune responses, and microbial dysbiosis constituting key determinants in the disease’s pathological progression (Teles et al., 2022; Di Stefano et al., 2022; Darby, 2022). Specifically, genetic predisposition may make some individuals less effective in the immune response to bacterial infection, and thus more prone to inflammation (Laine et al., 2012). At the same time, if the bacterial community in the mouth is unbalanced, the excessive growth of certain pathogenic bacteria will also exacerbate the inflammatory response (Bendek et al., 2021). Bacterial invasion can cause a local immune response, leading to an inflammatory response that further destroys periodontal tissue, which can eventually lead to tooth loosening, tooth loss, and even affect overall health, enhancing predisposition to chronic pathological conditions, particularly cardiovascular disorders and metabolic syndromes including diabetes mellitus. In addition to genetic factors, external determinants including tobacco consumption, inadequate dental care maintenance, suboptimal dietary patterns, and underlying medical conditions can also significantly increase the risk of periodontitis (Genco and Borgnakke, 2013; Bartold, 2018).

Periodontitis is usually caused by plaque buildup, which leads to an inflammatory response in the gums and periodontal tissues. The symptoms of early periodontitis are mild and may be manifested as bleeding gums, bad breath and swollen gums (Teles et al., 2022; Heitz-Mayfield, 2024). These symptoms are often mistaken for general gum problems and therefore easily overlooked or delayed treatment. However, if left untreated, the disease can progress to the middle and late stages, leading to serious destruction of periodontal tissue and even tooth loosening or loss. The treatment of advanced periodontitis is difficult and requires comprehensive treatment such as deep cleaning and surgical repair, and the risk of disease recurrence is high. With the objective of improving long-term patient outcomes and disease management, some new treatment methods, such as laser therapy and antimicrobial therapy, have been applied in clinical practice in recent years, and have achieved certain curative effects (Graziani et al., 2017; Kwon et al., 2021; Herrera et al., 2022; Cobb, 2017). However, there are risks such as high cost, strict technical requirements, risk of drug resistance and side effects, and the early diagnosis and treatment of periodontitis still face great challenges due to the lack of obvious early symptoms and specific biological markers. This study attempts to find key genes related to periodontitis by integrating microarray data and using random forest and WGCNA algorithms to provide reliable scientific basis for its early diagnosis and treatment targets.

## 2 Material and methods

### 2.1 Methods for data processing and software tools

The primary genomic data were acquired from the publicly accessible Gene Expression Omnibus (GEO) repository (Clough et al., 2024), including transcriptome gene expression matrix, platform information, and clinical information. This study included two datasets: GSE10334 and GSE16134, which were divided into the periodontitis gingival affected group and the periodontitis gingival unaffected group.

All computational procedures and statistical evaluations were performed utilizing the R programming platform (v4.4.0), maintained by the R Development Core Team. This open-source statistical software package can be accessed through its official web portal at https://www.r-project.org/ (Ariel de Lima et al., 2022). To address potential batch effects across multiple datasets, we implemented the ComBat normalization method implemented in the sva package of the R programming environment. This methodology, grounded in empirical Bayesian statistical principles, was implemented to address batch-related variations and ensure dataset consistency (Leek et al., 2012). The ComBat approach adjusts for systematic differences between batches by utilizing a Bayesian framework to ensure that data from different batches is comparable for downstream analysis. This method can effectively correct batch effects caused by technical or experimental factors in the data, thereby reducing the interference of these non-biological variations on the results. Transcriptomic profiling and identification of differentially expressed genes (DEGs) were conducted utilizing the limma computational tool within the R statistical environment, which implements an empirical Bayesian approach for analyzing microarray data. This well-established statistical framework enables robust detection of differentially expressed genes while controlling for multiple testing (Ritchie et al., 2015). To visualize transcriptional variations, hierarchical clustering was conducted through the pheatmap visualization tool implemented in the R programming framework. This package implements robust clustering algorithms and color normalization methods to generate comprehensive heatmap visualizations, enabling effective pattern recognition and biological interpretation of gene expression profiles. To systematically investigate inter-gene co-expression relationships and identify functional gene modules, We utilized the WGCNA computational framework, which was executed within the R programming environment. This comprehensive systems biology approach utilizes soft-thresholding and topological overlap matrix calculations to construct scale-free gene co-expression networks, facilitating the detection of functionally relevant gene clusters and key regulatory elements correlated with distinct phenotypic characteristics (Langfelder and Horvath, 2008). When screening for key genes, we also used the “randomForest” R package (Nguyen et al., 2021), a powerful machine learning tool to implement a decision tree and assess the relative contribution of each gene to screen out the key genes that are most relevant for disease. Upon detection of pivotal candidate genes, pathway enrichment investigations were conducted utilizing the clusterProfiler computational tool to characterize associated biological mechanisms and molecular networks. This comprehensive analysis incorporated Gene Ontology (GO) enrichment across three domains (biological process, molecular function, and cellular component) and pathway analysis using the Kyoto Encyclopedia of Genes and Genomes (KEGG) database, with false discovery rate (FDR) correction applied to ensure statistical rigor (Wu et al., 2021). The fundamental biological mechanisms, molecular interactions, and cellular signaling cascades associated with pivotal genes were systematically investigated.

### 2.2 WGCNA analysis

To investigate gene co-expression patterns associated with disease pathogenesis, we implemented a comprehensive analytical pipeline using the WGCNA framework. Prior to network construction, rigorous quality assessment protocols were implemented to ensure data integrity and analytical robustness. Specifically, we performed systematic data preprocessing, including calculation of pairwise Pearson correlation coefficients (PCC) across all gene pairs to assess expression similarity. Potential outliers were identified through robust statistical measures, including interquartile range (IQR) analysis and Mahalanobis distance calculation, with stringent filtering criteria applied to maintain dataset consistency and reliability. In order to further establish a scale-free network of biological significance, we selected an appropriate soft threshold parameter, set to 0.9, which enables us to screen out the most relevant nodes in the gene expression profile. Based on the soft threshold, we successfully constructed the co-expression relationship between genes, and by using this standard, genes were classified into several different modules according to their expression patterns. Each co-expression module comprises a functionally coherent gene cluster exhibiting highly correlated expression profiles, suggesting potential synergistic involvement in specific biological processes or pathways. These modules were characterized by distinct topological properties and intramodular connectivity patterns, reflecting their potential roles in coordinated biological functions and regulatory mechanisms. Finally, we focus on the identification of key genes in the network, namely, hub genes. These hub genes serve pivotal functions in the network and are often closely related to the occurrence and development of diseases. Therefore, we are focusing on these hub genes as the focus of subsequent studies to further explore their potential functions and regulatory roles in disease mechanisms.

### 2.3 Random forest

In this study, we combine two independent data sets to form a new representation matrix, and perform differential representation analysis on this matrix. The analysis process used the “limma” R package, a commonly used differential analysis tool that enables efficient processing of gene expression data. We then selected the most significant genes in the differential expression analysis, including the 300 most significantly upregulated and 300 most significantly downregulated differentially expressed genes. These genes will be used as input data for subsequent analysis and random forest analysis will be performed using the “randomForest” R package. This algorithm demonstrates exceptional performance in handling high-dimensional datasets, particularly due to its inherent regularization mechanisms that effectively mitigate overfitting. The robustness of the model is further enhanced by its capacity to maintain optimal bias-variance tradeoff, even when processing datasets with feature dimensions significantly exceeding sample size. In the process of model training, the random forest generates multiple decision trees by randomly partitioning the data set. These trees are each trained with different data subsets and feature subsets, so they can capture diverse features in the data and improve the accuracy of the prediction (Dunne et al., 2023; Quadrianto and Ghahramani, 2015). After the training is complete, the random forest model possesses the capability to quantitatively assess and rank the relative importance of individual predictor variables in determining the final prediction outcomes (that is, each gene) in the overall model. This importance value is usually measured by the “Gini index,” which reflects the relative importance of individual features in determining model classification outcomes (Wang et al., 2016). Based on the feature importance scores derived from the Gini index analysis, we systematically ranked all genes and subsequently identified the top-ranking candidates as potential key genes.

### 2.4 GO and KEGG analysis

We pooled 24 differential genes obtained from random forest and 65 differential genes obtained from WGCNA for downstream functional enrichment analysis. GO term enrichment analysis aims to reveal the multi-dimensional roles of genes in biological systems through functional annotation of gene sets. The Gene Ontology (GO) analysis framework comprises three fundamental aspects: Molecular Function (MF), addressing elemental activities at the molecular level; Biological Process (BP), describing coordinated cellular events; and Cellular Component (CC), characterizing subcellular structures and locations. Molecular function describes the specific functions performed by gene products at the molecular level, biological processes encompass a diverse array of molecular interactions and cellular events mediated by gene products, and cellular components describe the specific location or subcellular structure of gene products in cells. To perform Go-related enrichment analyses, we use the “enrichKEGG” R package (Wu et al., 2021). The package was able to identify statistically significant GO terms that were closely related to the gene sets provided, which in turn helped us discover and to elucidate molecular mechanisms and regulatory functions mediated of these genes defined by GO classification. Through this approach, we systematically detected statistically significant alterations across multiple ontological categories, including key biological pathways, molecular interaction networks, and subcellular localization patterns associated with the gene set under investigation, providing valuable insights into their potential biological significance. Furthermore, to comprehensively investigate the functional characteristics of these genes, we employed the “enrichKEGG” R package, a bioinformatics tool that integrates with the Kyoto Encyclopedia of Genes and Genomes (KEGG) database, enabling systematic analysis of the biological pathways and molecular networks associated with these genes (Wu et al., 2021). KEGG pathway analysis can reveal metabolic pathways, signal transduction pathways, and other important biological networks related to study genes. This analysis helps us understand the functional location of genes in cells and their roles in various biological processes, further enhancing our understanding of their underlying mechanisms and interactions.

### 2.5 Ethical declaration

The research data utilized in this investigation were exclusively obtained from open-access repositories and established public domain resources, and we relied entirely on existing, publicly available datasets without conducting any experiments or interventions directly related to individual animals or humans. Therefore, this study does not require the participation of animal experiments or human subjects and fully complies with all ethical guidelines and regulations.

## 4 Results

### 4.1 Data details

This investigation incorporated two independent datasets, with their comprehensive characteristics and experimental parameters systematically summarized in Table1.

**TABLE 1:** Summary of dataset characteristics and experimental parameters.

GSE ID	Partipants	Species	Analysis type	Year
GSE10334	183 periodontitis and 64 healthy	Human	Array	2008
GSE36090	241 periodontitis and 69 healthy	Human	Array	2009

### 4.2 Research workflow and methodological framework

**FIGURE 1:**
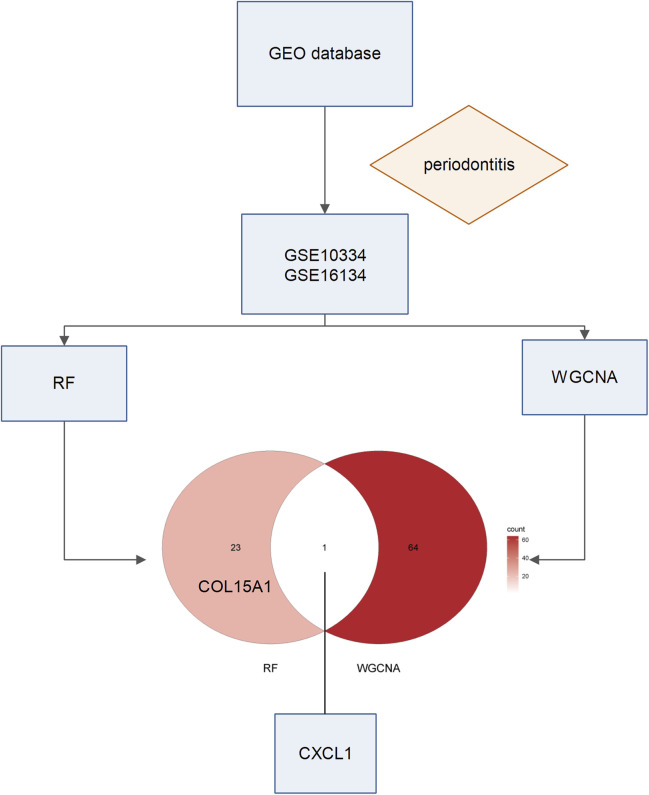
Workflow of this study. We first conducted an extensive search in the GEO database, aiming to find a suitable dataset related to periodontitis. After screening, we finally selected two datasets, GSE10334 and GSE16134. Next, we conducted RF analysis and WGCNA on both datasets. By integrating the results from these two approaches, we identified a potential key gene.

The present investigation involved the acquisition and integration of two distinct gene expression datasets from the GEO repository. Subsequent analytical procedures incorporated both RF algorithms and WGCNA methodologies to systematically examine the combined dataset characteristics. The analytical pipeline yielded distinct sets of DEGs through complementary approaches: WGCNA methodology identified 65 significant gene candidates, while the RF-based classification algorithm detected 24 potential biomarkers, demonstrating partial overlap between the two analytical frameworks. Finally, one gene CXCL1 was obtained. COL15A1 and CTSH ranked first in the results of random forest analysis, and MICU3 and EIF3D ranked first in the results of WGCNA analysis. Therefore, we take these five genes as the key genes, and see Figure 1 for the specific process.

### 4.3 Data normalization and preprocessing pipeline

**FIGURE 2:**
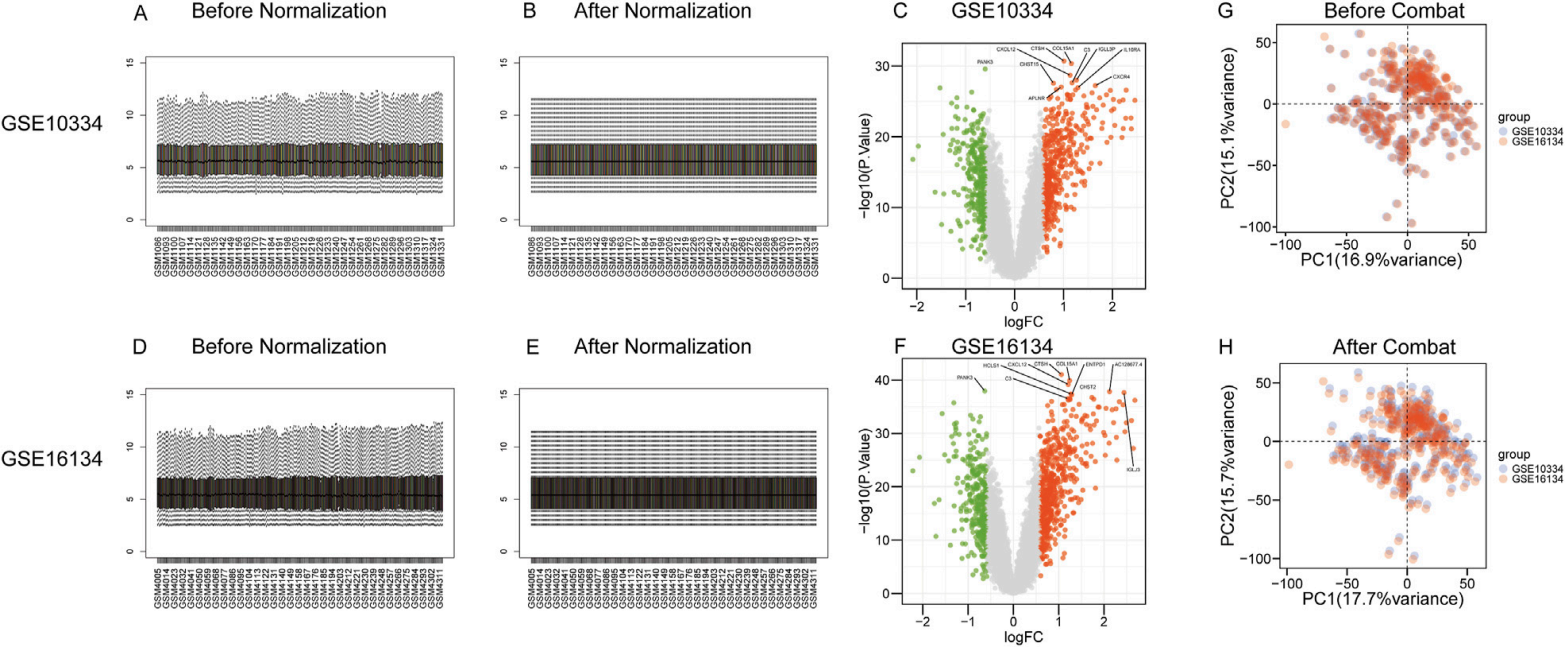
Data preprocessing and integration. First, we use the limma software package to standardize a single dataset. **(A, D)** is the distribution of data before standardization, and **(B, E)** is the distribution of data after standardization. **(C, F)** Two datasets were analyzed for difference, and the ten genes that had the most significant P - values were marked. To mitigate the potential confounding effects of batch processing on analytical outcomes, we utilize the Combat function which is included in the “sva” R package. The Combat function minimizes the interference of these batch effects on data results by adjusting and removing variations caused by batch-specific factors. **(G)** The distribution of the initial data set before the batch effect is removed, and **(H)** the distribution of the data set after the batch effect is removed.

The microarray datasets were initially processed using quantile normalization to standardize expression values across all samples. The result is Figures 2A, B, D, E. We carried out difference analysis on the two datasets respectively, and labeled the top 10 most significant genes in p-values, as shown in Figures 2C, F. For comprehensive data harmonization, including noise reduction, redundancy elimination, and batch effect adjustment, we applied the ComBat normalization method available in the sva R package. By removing the batch effect, we verified that the corrected data set had a consistent expression pattern across batches and retained true biological differences, as shown in Figures 2G, H.

### 4.4 Network topology and module identification through WGCNA

**FIGURE 3:**
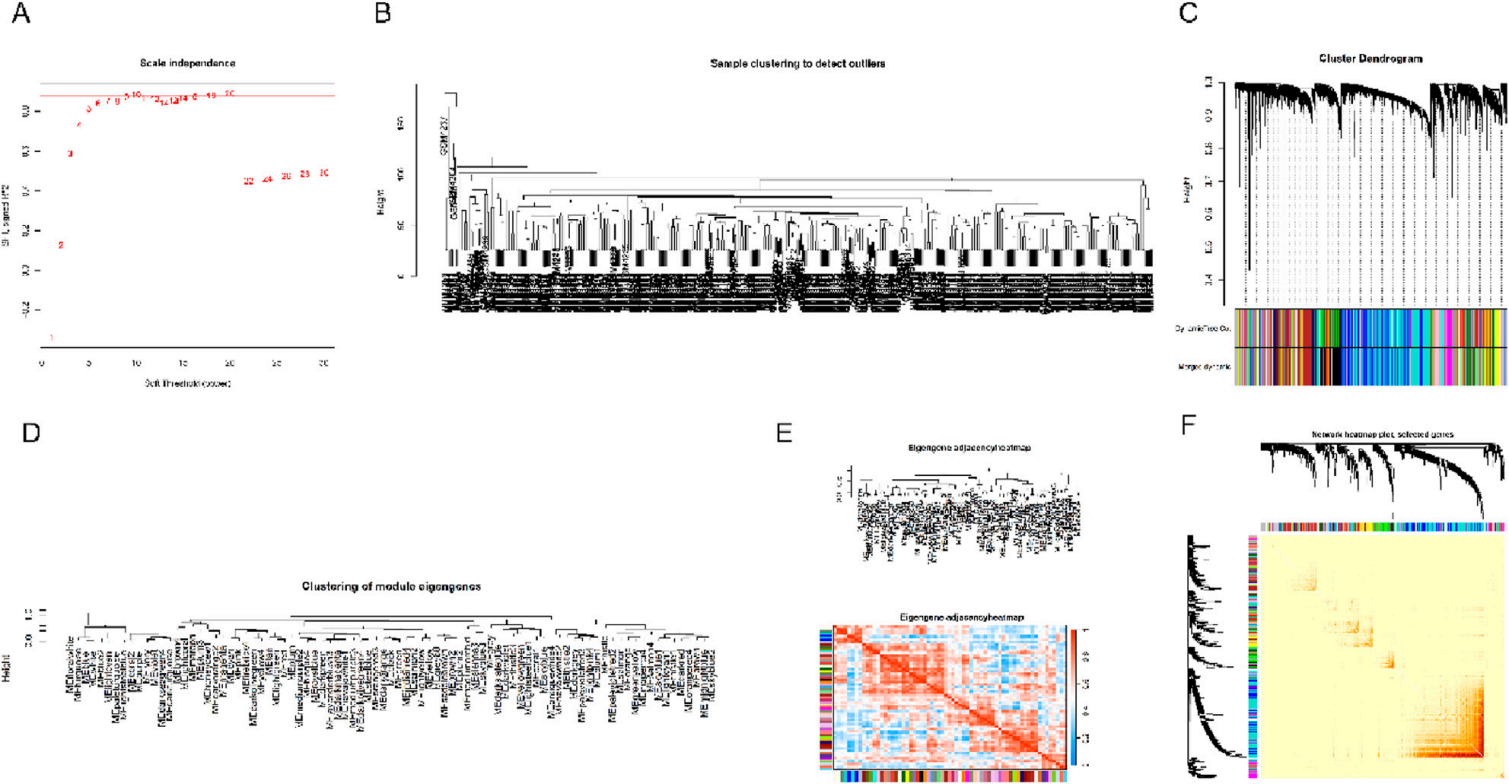
Results of the WGCNA analysis. **(A)** In the process of WGCNA analysis, select appropriate soft threshold parameters to ensure the accuracy of network construction. **(B–E)** These diagrams show the process of gene module identification and characterization under the WGCNA framework. Specifically, they show how a gene can be rationally divided into different co-expression modules based on its expression profile. Gene co-expression modules represent clusters of genes demonstrating coordinated transcriptional profiles, indicating potential functional associations and participation in shared biological pathways. **(F)** The figure further illustrates how the entire gene expression matrix is divided into these identified modules, showing how the data is segmented into several biologically relevant clusters.

As a sophisticated computational framework, WGCNA has emerged as a powerful methodology in biomedical investigations, enabling the systematic construction of gene interaction networks and identification of functionally relevant modules associated with pathological conditions. This analytical approach facilitates the discovery of molecular signatures, elucidation of disease pathogenesis, and identification of novel therapeutic interventions through comprehensive network-based analyses. The foundational concept underlying WGCNA methodology revolves around the systematic examination of pairwise gene expression correlations, cluster genes into distinct co-expression modules and subsequently identify disease-relevant modules through comprehensive network analysis. The identified co-expression modules represent functionally coherent gene clusters that demonstrate consistent expression patterns across biological conditions, providing valuable insights into potential disease-associated biomarkers. By implementing a network-based analytical framework, WGCNA facilitates the comprehensive detection and characterization of highly interconnected hub genes within complex gene regulatory architectures - highly interconnected nodes that frequently serve as critical regulators in disease pathogenesis and progression. This analytical approach not only facilitates the elucidation of disease mechanisms at the molecular level but also establishes a robust framework for biomarker discovery and the design and implementation of precision-based treatment strategies tailored to specific molecular targets and pathological mechanisms. To comprehensively investigate the systemic impact of periodontitis on physiological functions, we employed WGCNA to analyze the periodontitis-associated gene expression matrix. While numerous studies have explored individual gene functions in periodontitis, substantial limitations persist in the comprehensive elucidation of intricate transcriptional regulatory mechanisms and their multidimensional interactions within cellular systems and their interactions underlying this condition. Our network-based approach provides a systems-level perspective that complements traditional gene-centric analyses, potentially revealing novel molecular pathways and therapeutic targets in periodontitis. To maintain analytical rigor and ensure the reliability of our findings, we first used the “goodSamplesGenes ()” function to check the quality of sample data and remove the outliers in the data. Then, the “hclust()” function is used to perform cluster. To establish an optimal scale-free network topology, the optimal soft-thresholding parameter was determined through systematic computational analysis using the pickSoftThreshold() algorithm, with selection criteria incorporating both scale-free topology approximation (minimum R^2^ = 0.85) and network connectivity preservation. Following network construction, we performed hierarchical clustering of the topological overlap matrix (TOM) and implemented a dynamic tree-cutting algorithm with a deepSplit parameter of 3 and minimum module size of 40 genes, ensuring biologically meaningful module detection while preventing excessive fragmentation. In order to identify and visualize different modules, we use the “labels2colors()” function to represent each module with a different color. The correlation between the different modules is then calculated using the “moduleEigengenes()” function, helping us to further understand the relative relationship of these modules in the overall gene network. During the final stage of network analysis, genes demonstrating ≥90% topological overlap similarity were clustered into cohesive modules. Subsequent identification of intramodular hub genes, characterized by their high connectivity and module membership scores, revealed critical regulatory nodes that provide mechanistic insights for further functional investigation. The relevant results of this process have been shown in Figure 3, further revealing the possible role of periodontitis-related genes in the physiological and pathological processes of the body.

### 4.5 Results of the RF analysis

**FIGURE 4:**
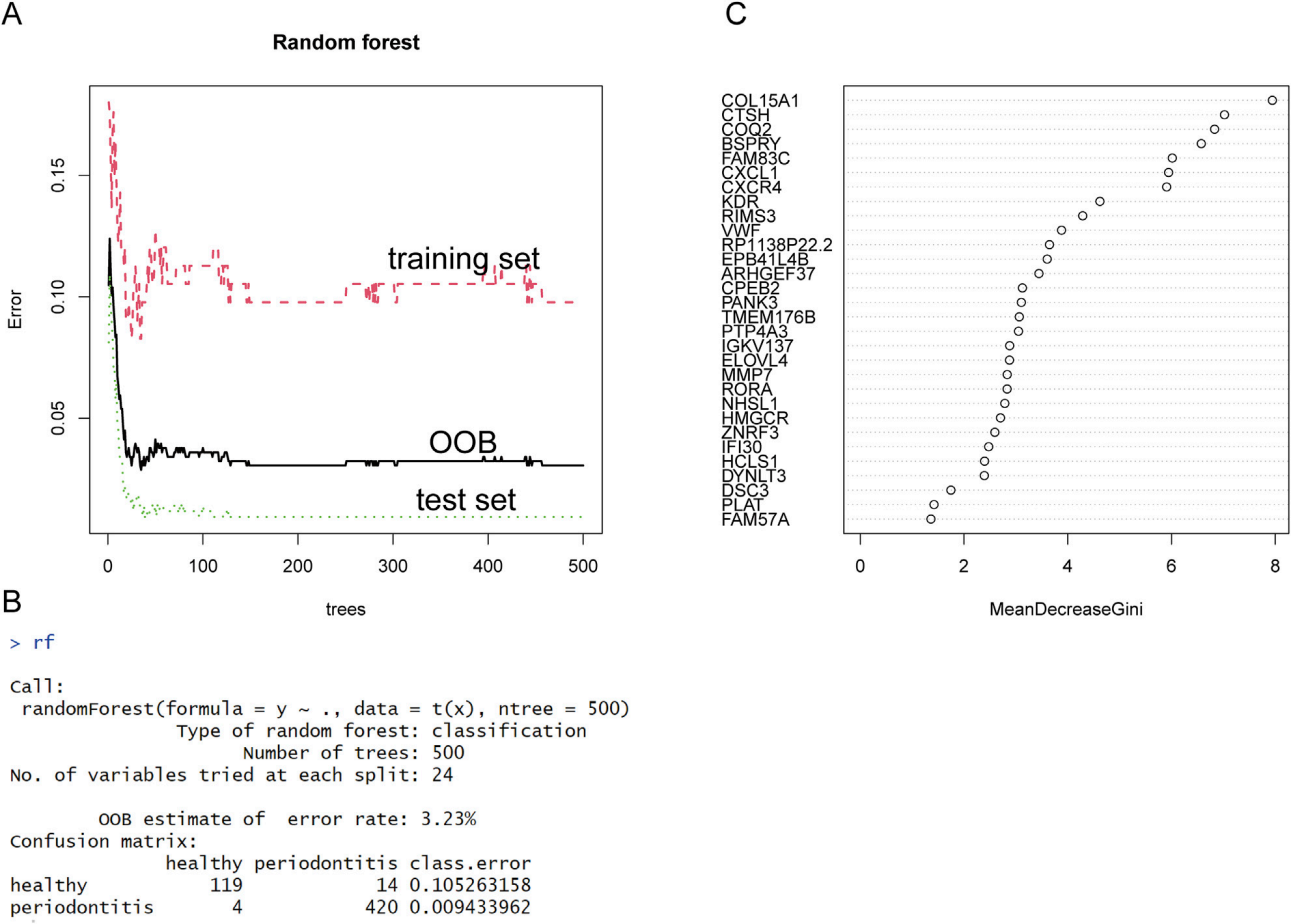
Results of the RF analysis. **(A)** The error rates of training set, test set and OOB gradually decrease with a growing number of trees. **(B)** When the OOB error rate tends to be stable, the number of trees is 24, and the error rate is 3.23%. **(C)** We then used the “Gini Index” as an evaluation indicator to show the importance ranking of each gene.

As an ensemble learning methodology, Random Forest (RF) has emerged as a robust computational framework extensively applied in biomedical informatics and computational biology research domains, especially in the screening of disease-related genes. In disease research, random forest can effectively screen out key genes that are closely related to disease from large amounts of genetic data. Compared with the traditional single-variable analysis method, random forest can handle high-dimensional data and complex gene interactions by integrating multiple decision trees, and it is not easy to overfit. In this study, we combine two independent data sets into a new representation matrix, and analyze the differential representation of this matrix. We then selected the most significant genes in the differential expression analysis, including the 300 most significantly upregulated and 300 most significantly downregulated DEGs. These genes will be used as input data for subsequent analysis. To ensure robust model validation, we performed stratified random partitioning of the identified gene set into independent training and testing cohorts. As the number of decision trees increases, the OOB error rate decreases. When the OOB error rate is 3.23%, it means that the model has converged to A relatively stable state, and the number of decision trees is 24. See Figures 4A, C for specific results. According to the feature importance score calculated by the Gini index, we ranked all the genes and screened out the top 24 genes as potential key genes in Figure 4B.

### 4.6 Results of the GO and KEGG analysis

**FIGURE 5:**
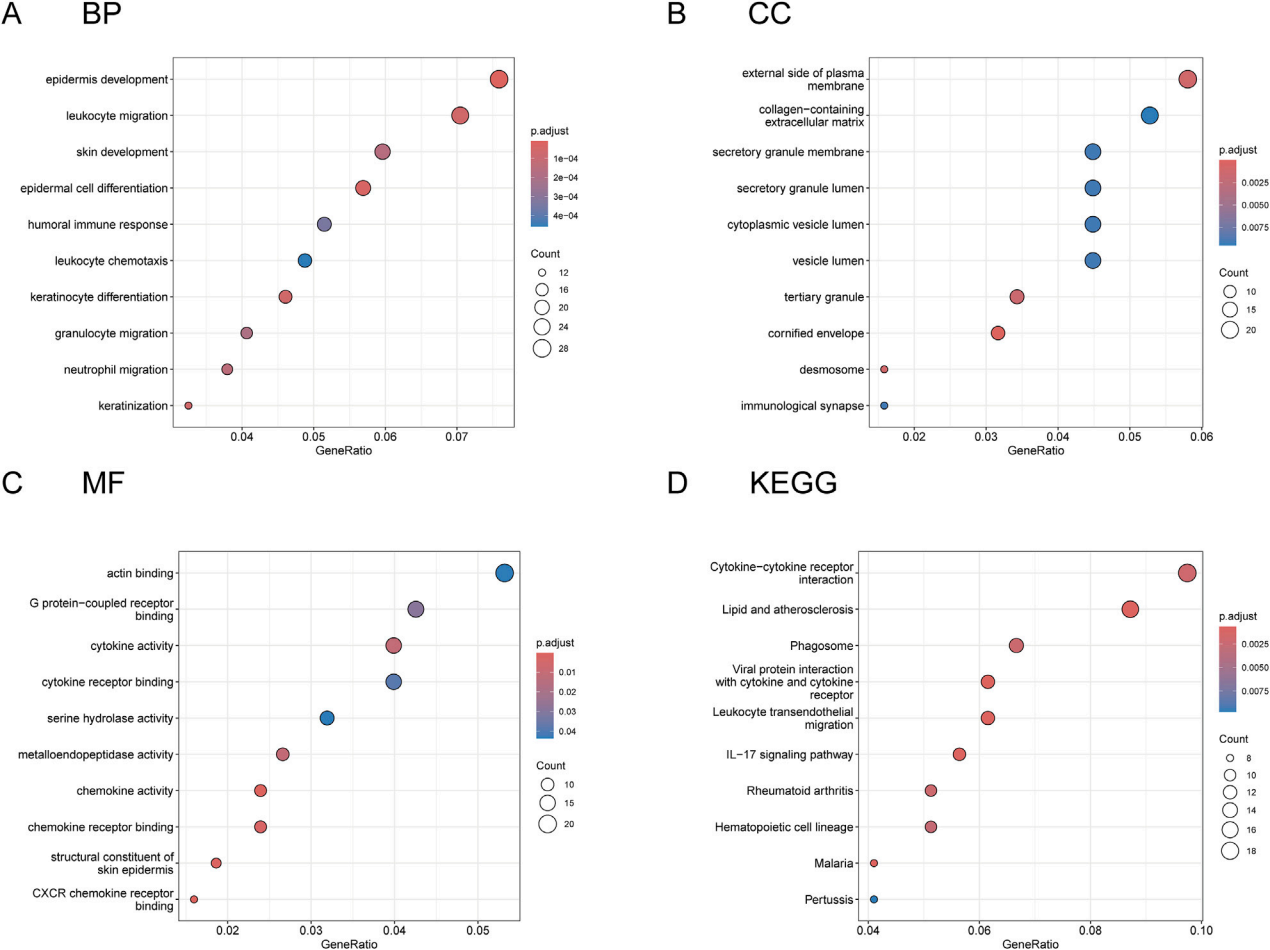
The GO and KEGG pathway analyses of the combined DEGs. Through the integration of RF machine learning algorithms and WGCNA, we delineated a distinct cohort of differentially expressed genes. Subsequent functional annotation of these molecular signatures was conducted via comprehensive Gene Ontology (GO) enrichment analysis. **(A–C)** Displays the top 10 enriched pathways categorized by the three primary GO domains: biological process (BP), cellular component (CC), and molecular function (MF). These pathways reflect the functional characteristics and activities of genes involved in various biological processes, their cellular locations, and their molecular functions. **(D)** Delineates the ten most significantly enriched pathways derived from KEGG pathway enrichment analysis, elucidating potential molecular mechanisms and biological processes associated with the differentially expressed gene clusters.

In the following analysis, we pooled 24 differentially expressed genes (DGEs) identified by random forest (RF) analysis with 65 DEGs screened from WGCNA. With this approach, we get a comprehensive gene set. To investigate the functional relevance of the identified gene clusters, a multi-dimensional annotation approach was implemented. Gene Ontology (GO) term enrichment analysis was systematically conducted, encompassing biological processes, molecular functions, and cellular components. In parallel, pathway enrichment profiling was performed through the KEGG platform to detect statistically significant pathway alterations and molecular network perturbations. The comprehensive outcomes of our comprehensive functional annotation and pathway enrichment investigations are systematically presented in Figure 5, which illustrates the significant associations between DEGs and specific biological processes. The visualization reveals distinct patterns of gene enrichment across various functional categories and metabolic pathways, highlighting their potential regulatory roles in key cellular processes. Notably, the network diagram demonstrates the complex interplay between significantly enriched pathways and their associated gene clusters, yielding critical mechanistic understanding of the molecular pathways and regulatory networks associated with the identified phenotypic alterations. GO analysis results showed that the first enrichment result in BP was epidermis development. In CC, the first enrichment result is the external side of plasma membrane. In MF, the first priority of enrichment result is actin binding, while in KEGG enrichment result, the first priority is Cytokine-cytokine receptor interaction.

## 5 Discussion

Our study aims to explore potential biological targets of periodontitis through in-depth analysis of 2 independent data sets. Through the integration of diverse analytical approaches, notably the RF machine learning algorithm and WGCNA, we have successfully identified critical genetic markers and pivotal biological pathways associated with periodontitis pathogenesis. These significant findings not only enhance our mechanistic understanding of periodontitis at the molecular level but also establish a foundation for exploring novel therapeutic targets and advancing innovative treatment strategies for this prevalent oral disease.

CXCL1 (C-X-C Motif Chemokine Ligand 1) is a protein-coding gene. CXCL1 is a chemokine belonging to the CXC subfamily. Also known as GROα (Growth-Regulated Oncogene Alpha). The main role is to modulate the chemotactic migration and functional activation of immunocompetent cells by binding to its receptors (such as CXCR2) (Korbecki et al., 2022a). CXCL1 serves as a pivotal mediator in orchestrating diverse immune responses and inflammatory cascades. As a potent chemokine, its fundamental biological function involves the recruitment and directional migration of immunocompetent cells, particularly neutrophils, to localized sites of infection or tissue inflammation through chemotactic signaling. Through its specific binding to the CXCR2 receptor, CXCL1 initiates a cascade of intracellular signal transduction pathways that regulate essential cellular processes, including directional cell migration, functional activation of immune cells, and the modulation of inflammatory responses (Korbecki et al., 2022b; Silva et al., 2017). Specific functions of CXCL1 include: 1. Recruitment of immune cells: CXCL1 attracts neutrophils to inflammatory or infected areas through its binding with CXCR2 receptors to enhance immune response (Girbl et al., 2018; De Filippo et al., 2013). 2. Proinflammatory effect: CXCL1 can activate immune cells and enhance their ability to kill bacteria and remove pathogens (Wang et al., 2022; Kaltenmeier et al., 2022). 3. Tissue repair: In some cases, CXCL1 also plays a role in the repair process after tissue injury, especially in cell migration and new blood vessel formation (Kaur et al., 2023; Wang et al., 2018). CXCL1 has been extensively implicated in the molecular pathophysiology of diverse disease states, particularly through its critical involvement in inflammatory cascades, immune diseases and tumors. Due to its role in promoting immune cell migration and activation, emerging evidence has established CXCL1 as a pivotal molecular regulator in the development and progression of various inflammatory pathologies, with demonstrated involvement in periodontal disease, autoimmune arthritis, and chronic intestinal inflammation, among other immune-mediated conditions (Hou et al., 2023; Liu W. et al., 2018; Zhan et al., 2023). The investigation revealed significantly elevated CXCL1 expression levels in gingival tissues obtained from both human subjects and rat models with periodontitis, when compared to healthy periodontal sites (Rath-Deschner et al., 2020). Enhancement of CXCL1 expression in gums has been reported to normalize diabetes - and insulin-resistant induced neutrophil recruitment and delayed periodontitis. Therapeutic modulation of CXCL1 dysregulation in fibroblasts represents a promising strategy not only for periodontitis management but also for ameliorating diabetes-related complications, including insulin resistance and impaired wound healing (Shinjo et al., 2023). In another work integrating the single-cell transcriptome, it was found that CXCL1 inhibited by curcumin could exert therapeutic potential in the management of periodontitis (Huang et al., 2023). CXCL1 has been mechanistically linked to tumorigenesis and metastatic progression across multiple malignancies, with well-documented roles in non-small cell lung carcinoma, mammary neoplasia, pancreatic adenocarcinoma, and cutaneous melanoma pathogenesis. Emerging evidence highlights the pivotal role of CXCL1 in driving oncogenic processes and promoting the metastatic spread of malignant cells to secondary sites through dual mechanisms: orchestrating the recruitment and accumulation of immunosuppressive cells within the tumor microenvironment, and enhancing the invasive potential and metastatic capabilities of malignant cells (Lu et al., 2024; Yu et al., 2019; Liang et al., 2021; Lv et al., 2014; Zheng et al., 2023; Hayashi et al., 2023; Molinelli et al., 2023). CXCL1 has been mechanistically linked to the pathogenesis of cardiovascular disorders, particularly atherosclerosis and coronary artery disease, through its ability to exacerbate vascular inflammation by promoting pro-inflammatory responses within the vascular endothelium (Korbecki et al., 2022c; Baragetti et al., 2023). According to previous evidence, CXCL1 does participate in the occurrence of periodontitis, which further validates the feasibility and scientificity of our algorithm.

The COL15A1 gene encodes the α1 subunit of type XV collagen, a distinctive member of the FACIT collagen family characterized by its unique structural organization featuring intermittent triple-helical domains (Eklund et al., 2000; Hagg et al., 1998). Type XV collagen exhibits ubiquitous expression across multiple tissue types, but the predominant localization of this protein within the basement membrane zone suggests its potential involvement in mediating structural interactions between the basal lamina and subjacent stromal extracellular matrix. The proteolytic cleavage of type XV collagen generates a C-terminal fragment known as restin, which shares structural homology with endostatin and exhibits potential anti-angiogenic properties (Martinez-Nieto et al., 2021; Mutolo et al., 2012). Accumulating evidence from experimental studies has demonstrated that genetic deficiency of collagen XV is associated with progressive muscular degeneration and microvascular abnormalities (Schupp et al., 2023). COL15A1 serves a critical structural and functional role in maintaining the integrity of ocular basement membranes, particularly in corneal tissues. Genetic alterations in COL15A1 have been implicated in the pathogenesis of various ocular disorders, including keratopathy, visual dysfunction, and other sight-threatening conditions. Notably, emerging molecular epidemiology evidence demonstrates that polymorphic variants in COL15A1 and COL18A1 genes substantially modulate the temporal onset and clinical trajectory of primary open-angle glaucoma (POAG) (Wiggs et al., 2013). Due to the expression of COL15A1 in the heart and blood vessels, its mutation may be associated with heart disease, especially with abnormal myocardial morphology and physiology (Durgin et al., 2017; Grimaldi et al., 2015). It was found that overexpression of downregulated Col15a1 and increased the Col1a1/Col3a1 ratio. This mechanism may influence diastolic heart failure in diabetic cardiomyopathy by modulating myocardial stiffness and elasticity (Dai et al., 2024). Another study found that Col15a1, a vascular secretory factor secreted by coronary arteries, is downregulated by Ino80 defect and actively facilitates cardiomyocyte proliferation (Rhee et al., 2021). Abnormalities in COL15A1 may also play a role in certain connective tissue diseases, which modulates tissue architecture and physiological functionality such as skin, bone and muscle (Gabusi et al., 2012), for instance, a study revealed that both the loss and gain of col15a1b function result in pathfinding errors in primary and secondary motor neuron axons. Pathfinding abnormalities manifest both at and distal to the critical decision point where axonal trajectory determination occurs, ultimately resulting in progressive muscular degeneration and impaired locomotor function (Guillon et al., 2016). Additional studies have demonstrated that col15a1b deficiency impairs peripheral nerve maturation, resulting in structural abnormalities of the basal lamina and consequent impairment of sensorimotor integration processes (Rasi et al., 2010). Another study found that COL15A1 is a candidate gene for further studies to evaluate the genetic susceptibility to osteoporosis (Trost et al., 2010). Moreover, Col15a1 has been implicated in tumorigenesis. Studies have shown that Col15a1 inactivation in mice alters the fibrotic tumor microenvironment and promotes breast tumor progression (Martinez-Nieto et al., 2021). Emerging research evidence indicates that elevated expression levels of COL15A1 significantly inhibit the migratory capacity and metastatic potential of testicular seminoma cells (Cui et al., 2021). Genome-wide association analyses have revealed COL15A1 as a novel genetic susceptibility locus associated with epithelial ovarian cancer risk in the Chinese Han population (Chen et al., 2014).

We identified two key genes significantly associated with the progression of periodontitis, which play a critical role in its pathogenesis. Existing research indicates that these genes are not only closely linked to periodontitis but also implicated in various inflammatory diseases, tumors, and cardiovascular disorders. The experimental evidence implies a potential etiological role of these genetic factors in the pathogenesis and progression of periodontal disease. From a clinical standpoint, the expression profiles of these genes present valuable biomarkers with considerable diagnostic and therapeutic implications. Tracking the expression patterns of these genes not only facilitates early disease detection but also enables accurate assessment of patient prognosis, thereby supporting the formulation of personalized treatment strategies. Importantly, the upregulation of certain genes is closely linked to adverse clinical outcomes, making them reliable predictors for survival rates and recurrence probabilities. Consequently, analyzing the expression dynamics of these genes offers critical insights for clinical decision-making, aids in the timely identification of early-stage periodontal disease markers, and plays a pivotal role in optimizing disease management protocols. Further, previous translational medicine research has shown that some key genes have the prospect of being potential targets in clinical therapy. By inhibiting or activating these genes, we can interfere with the proliferation of cells or regulate the inflammatory response, thereby optimizing therapeutic outcomes and ameliorating clinical manifestations in affected individuals. The detection technology of these genes can not only act as a pivotal modulator of patient screening, but also assist clinicians in selecting the most optimal treatment strategy, thereby improving the accuracy and effectiveness of treatment. In the future, a deeper exploration of the functional roles of these critical genes will provide valuable insights into their molecular mechanisms underlying the development and progression of periodontitis. By clarifying the precise contributions of these genes, this research will accelerate the development of novel diagnostic tools and precision-based therapeutic strategies. Additionally, by utilizing the expression patterns of these genes, scientists can further explore their clinical applications, particularly in areas such as early disease detection, prognosis assessment, and the prediction of therapeutic outcomes. Future multi-center clinical studies will help verify the clinical application value of these genes in periodontitis, and provide more comprehensive support for precision medicine of periodontitis through the combination of multi-dimensional data such as genomics and proteomics. This will not only promote the progress of early diagnosis and accurate treatment of periodontitis, but also lay a solid foundation for the implementation of personalized medicine.

### 5.1 Limitation

The main limitation of this study is that the data set is relatively small, which may cause some deviations in the results. We have searched the GEO database for microarray data on both affected and unaffected gums in all patients with PD, unfortunately, we found only two datasets that met the inclusion criteria, which were compensated for by a sufficient sample size. In future studies, we will validate our results in other ways.

## Data Availability

The data presented in the study are deposited in the GEO repository, accession number GSE10334, GSE36090.

## References

[B1] Ariel de Lima D. Helito C. P. de Lima L. L. Clazzer R. Goncalves R. K. de Camargo O. P. (2022). How to perform a meta-analysis: a practical Step-by-Step guide using R software and Rstudio. Acta Ortop. Bras. 30 (3), e248775. 10.1590/1413-785220223003e248775 35694025 PMC9150877

[B2] Baragetti A. Da Dalt L. Moregola A. Svecla M. Terenghi O. Mattavelli E. (2023). Neutrophil aging exacerbates high fat diet induced metabolic alterations. Metabolism 144, 155576. 10.1016/j.metabol.2023.155576 37116643

[B3] Bartold P. M. (2018). Lifestyle and periodontitis: the emergence of personalized periodontics. Periodontol 78 (1), 7–11. 10.1111/prd.12237 30198129

[B4] Bendek M. J. Canedo-Marroquin G. Realini O. Retamal I. N. Hernandez M. Hoare A. (2021). Periodontitis and gestational diabetes mellitus: a potential inflammatory vicious cycle. Int. J. Mol. Sci. 22 (21), 11831. 10.3390/ijms222111831 34769262 PMC8584134

[B5] Chen K. Ma H. Li L. Zang R. Wang C. Song F. (2014). Genome-wide association study identifies new susceptibility loci for epithelial ovarian cancer in Han Chinese women. Nat. Commun. 5, 4682. 10.1038/ncomms5682 25134534

[B6] Clough E. Barrett T. Wilhite S. E. Ledoux P. Evangelista C. Kim I. F. (2024). NCBI GEO: archive for gene expression and epigenomics data sets: 23-year update. Nucleic Acids Res. 52 (D1), D138–D144. 10.1093/nar/gkad965 37933855 PMC10767856

[B7] Cobb C. M. (2017). Lasers and the treatment of periodontitis: the essence and the noise. Periodontol 75 (1), 205–295. 10.1111/prd.12137 28758295

[B8] Cui Y. Miao C. Liu S. Tang J. Zhang J. Bu H. (2021). Clusterin suppresses invasion and metastasis of testicular seminoma by upregulating COL15a1. Mol. Ther. Nucleic Acids 26, 1336–1350. 10.1016/j.omtn.2021.11.004 34853731 PMC8608570

[B9] Dai X. Yang F. Chen D. Yang L. Dong Z. Chen C. (2024). The role of fibromodulin in myocardial fibrosis in a diabetic cardiomyopathy rat model. FEBS Open Bio. 10.1002/2211-5463.13935 PMC11891772 39592912

[B10] Darby I. (2022). Risk factors for periodontitis and peri-implantitis. Periodontol 90 (1), 9–12. 10.1111/prd.12447 PMC9804916 35913624

[B11] De Filippo K. Dudeck A. Hasenberg M. Nye E. van Rooijen N. Hartmann K. (2013). Mast cell and macrophage chemokines CXCL1/CXCL2 control the early stage of neutrophil recruitment during tissue inflammation. Blood 121 (24), 4930–4937. 10.1182/blood-2013-02-486217 23645836

[B12] Del Pinto R. Ferri C. Giannoni M. Cominelli F. Pizarro T. T. Pietropaoli D. (2024). Meta-analysis of oral microbiome reveals sex-based diversity in biofilms during periodontitis. JCI Insight 9 (17), e171311. 10.1172/jci.insight.171311 39253976 PMC11385077

[B13] Di Stefano M. Polizzi A. Santonocito S. Romano A. Lombardi T. Isola G. (2022). Impact of oral microbiome in periodontal health and periodontitis: a critical review on prevention and treatment. Int. J. Mol. Sci. 23 (9), 5142. 10.3390/ijms23095142 35563531 PMC9103139

[B14] Dunne R. Reguant R. Ramarao-Milne P. Szul P. Sng L. M. F. Lundberg M. (2023). Thresholding Gini variable importance with a single-trained random forest: an empirical Bayes approach. Comput. Struct. Biotechnol. J. 21, 4354–4360. 10.1016/j.csbj.2023.08.033 37711185 PMC10497997

[B15] Durgin B. G. Cherepanova O. A. Gomez D. Karaoli T. Alencar G. F. Butcher J. T. (2017). Smooth muscle cell-specific deletion of Col15a1 unexpectedly leads to impaired development of advanced atherosclerotic lesions. Am. J. Physiol. Heart Circ. Physiol. 312 (5), H943-H958–H958. 10.1152/ajpheart.00029.2017 28283548 PMC5451587

[B16] Eke P. I. Wei L. Borgnakke W. S. Thornton-Evans G. Zhang X. Lu H. (2016). Periodontitis prevalence in adults ≥65 years of age, in the USA. Periodontol 72 (1), 76–95. 10.1111/prd.12145 PMC8223257 27501492

[B17] Eklund L. Muona A. Lietard J. Pihlajaniemi T. (2000). Structure of the mouse type XV collagen gene, Col15a1, comparison with the human COL15A1 gene and functional analysis of the promoters of both genes. Matrix Biol. 19 (6), 489–500. 10.1016/s0945-053x(00)00090-1 11068203

[B18] Gabusi E. Manferdini C. Grassi F. Piacentini A. Cattini L. Filardo G. (2012). Extracellular calcium chronically induced human osteoblasts effects: specific modulation of osteocalcin and collagen type XV. J. Cell Physiol. 227 (8), 3151–3161. 10.1002/jcp.24001 22034088

[B19] Genco R. J. Borgnakke W. S. (2013). Risk factors for periodontal disease. Periodontol 62 (1), 59–94. 10.1111/j.1600-0757.2012.00457.x 23574464

[B20] Girbl T. Lenn T. Perez L. Rolas L. Barkaway A. Thiriot A. (2018). Distinct compartmentalization of the chemokines CXCL1 and CXCL2 and the atypical receptor ACKR1 determine discrete stages of neutrophil diapedesis. Immunity 49 (6), 1062–1076. 10.1016/j.immuni.2018.09.018 30446388 PMC6303217

[B21] Graziani F. Karapetsa D. Alonso B. Herrera D. (2017). Nonsurgical and surgical treatment of periodontitis: how many options for one disease? Periodontol 75 (1), 152–188. 10.1111/prd.12201 28758300

[B22] Grimaldi V. Vietri M. T. Schiano C. Picascia A. De Pascale M. R. Fiorito C. (2015). Epigenetic reprogramming in atherosclerosis. Curr. Atheroscler. Rep. 17 (2), 476. 10.1007/s11883-014-0476-3 25433555

[B23] Guillon E. Bretaud S. Ruggiero F. (2016). Slow muscle precursors lay down a collagen XV matrix fingerprint to guide motor axon navigation. J. Neurosci. 36 (9), 2663–2676. 10.1523/JNEUROSCI.2847-15.2016 26937007 PMC6604867

[B24] Hagg P. M. Muona A. Lietard J. Kivirikko S. Pihlajaniemi T. (1998). Complete exon-intron organization of the human gene for the alpha1 chain of type XV collagen (COL15A1) and comparison with the homologous COL18A1 gene. J. Biol. Chem. 273 (28), 17824–17831. 10.1074/jbc.273.28.17824 9651385

[B25] Hayashi M. Ikenaga N. Nakata K. Luo H. Zhong P. Date S. (2023). Intratumor Fusobacterium nucleatum promotes the progression of pancreatic cancer via the CXCL1-CXCR2 axis. Cancer Sci. 114 (9), 3666–3678. 10.1111/cas.15901 37438965 PMC10475786

[B26] Heitz-Mayfield L. J. A. (2024). Conventional diagnostic criteria for periodontal diseases (plaque-induced gingivitis and periodontitis). Periodontol 95 (1), 10–19. 10.1111/prd.12579 38831568

[B27] Herrera D. Sanz M. Kebschull M. Jepsen S. Sculean A. Berglundh T. (2022). Treatment of stage IV periodontitis: the EFP S3 level clinical practice guideline. J. Clin. Periodontol. 49 (Suppl. 24), 4–71. 10.1111/jcpe.13639 35688447

[B28] Hewlett S. A. Anto F. Blankson P. K. Tormeti D. Ayettey-Adamafio M. Bayitse P. (2022). Periodontitis prevalence and severity in an African population: a cross-sectional study in the Greater Accra Region of Ghana. J. Periodontol. 93 (5), 732–744. 10.1002/JPER.21-0329 34724216

[B29] Holde G. E. Oscarson N. Trovik T. A. Tillberg A. Jonsson B. (2017). Periodontitis prevalence and severity in adults: a cross-sectional study in Norwegian circumpolar communities. J. Periodontol. 88 (10), 1012–1022. 10.1902/jop.2017.170164 28671509

[B30] Hou C. H. Chen P. C. Liu J. F. (2023). CXCL1 enhances COX-II expression in rheumatoid arthritis synovial fibroblasts by CXCR2, PLC, PKC, and NF-κB signal pathway. Int. Immunopharmacol. 124 (Pt B), 110909. 10.1016/j.intimp.2023.110909 37722260

[B31] Huang X. Liu Y. Wang Q. Rehman H. M. Horvath D. Zhou S. (2023). Brief literature review and comprehensive bioinformatics analytics unravel the potential mechanism of curcumin in the treatment of periodontitis. BMC Oral Health 23 (1), 469. 10.1186/s12903-023-03181-x 37422651 PMC10329799

[B32] Kaltenmeier C. Wang R. Popp B. Geller D. Tohme S. Yazdani H. O. (2022). Role of immuno-inflammatory signals in liver ischemia-reperfusion injury. Cells 11 (14), 2222. 10.3390/cells11142222 35883665 PMC9323912

[B33] Kaur G. Sharma D. Bisen S. Mukhopadhyay C. S. Gurdziel K. Singh N. K. (2023). Vascular cell-adhesion molecule 1 (VCAM-1) regulates JunB-mediated IL-8/CXCL1 expression and pathological neovascularization. Commun. Biol. 6 (1), 516. 10.1038/s42003-023-04905-z 37179352 PMC10183029

[B34] Kim H. S. Son J. H. Yi H. Y. Hong H. K. Suh H. J. Bae K. H. (2014). Association between harmful alcohol use and periodontal status according to gender and smoking. BMC Oral Health 14, 73. 10.1186/1472-6831-14-73 24950716 PMC4114163

[B35] Korbecki J. Barczak K. Gutowska I. Chlubek D. Baranowska-Bosiacka I. (2022a). CXCL1: gene, promoter, regulation of expression, mRNA stability, regulation of activity in the intercellular space. Int. J. Mol. Sci. 23 (2), 792. 10.3390/ijms23020792 35054978 PMC8776070

[B36] Korbecki J. Kupnicka P. Chlubek M. Goracy J. Gutowska I. Baranowska-Bosiacka I. (2022b). CXCR2 receptor: regulation of expression, signal transduction, and involvement in cancer. Int. J. Mol. Sci. 23 (4), 2168. 10.3390/ijms23042168 35216283 PMC8878198

[B37] Korbecki J. Maruszewska A. Bosiacki M. Chlubek D. Baranowska-Bosiacka I. (2022c). The potential importance of CXCL1 in the physiological state and in noncancer diseases of the cardiovascular system, respiratory system and skin. Int. J. Mol. Sci. 24 (1), 205. 10.3390/ijms24010205 36613652 PMC9820720

[B38] Kwon T. Lamster I. B. Levin L. (2021). Current concepts in the management of periodontitis. Int. Dent. J. 71 (6), 462–476. 10.1111/idj.12630 34839889 PMC9275292

[B39] Laine M. L. Crielaard W. Loos B. G. (2012). Genetic susceptibility to periodontitis. Periodontol 58 (1), 37–68. 10.1111/j.1600-0757.2011.00415.x 22133366

[B40] Langfelder P. Horvath S. (2008). WGCNA: an R package for weighted correlation network analysis. BMC Bioinforma. 9, 559. 10.1186/1471-2105-9-559 PMC2631488 19114008

[B41] Leek J. T. Johnson W. E. Parker H. S. Jaffe A. E. Storey J. D. (2012). The sva package for removing batch effects and other unwanted variation in high-throughput experiments. Bioinformatics 28 (6), 882–883. 10.1093/bioinformatics/bts034 22257669 PMC3307112

[B42] Liang Z. W. Ge X. X. Xu M. D. Qin H. Wu M. Y. Shen M. (2021). Tumor-associated macrophages promote the metastasis and growth of non-small-cell lung cancer cells through NF-κB/PP2Ac-positive feedback loop. Cancer Sci. 112 (6), 2140–2157. 10.1111/cas.14863 33609307 PMC8177805

[B43] Liu W. Zhang Y. Zhu W. Ma C. Ruan J. Long H. (2018b). Sinomenine inhibits the progression of rheumatoid arthritis by regulating the secretion of inflammatory cytokines and monocyte/macrophage subsets. Front. Immunol. 9, 2228. 10.3389/fimmu.2018.02228 30319663 PMC6168735

[B44] Liu Y. Yu Y. Nickel J. C. Iwasaki L. R. Duan P. Simmer-Beck M. (2018a). Gender differences in the association of periodontitis and type 2 diabetes. Int. Dent. J. 68 (6), 433–440. 10.1111/idj.12399 29786140 PMC9379021

[B45] Lu H. Ai J. Zheng Y. Zhou W. Zhang L. Zhu J. (2024). IGFBP2/ITGA5 promotes gefitinib resistance via activating STAT3/CXCL1 axis in non-small cell lung cancer. Cell Death Dis. 15 (6), 447. 10.1038/s41419-024-06843-y 38918360 PMC11199710

[B46] Lv M. Xu Y. Tang R. Ren J. Shen S. Chen Y. (2014). miR141-CXCL1-CXCR2 signaling-induced Treg recruitment regulates metastases and survival of non-small cell lung cancer. Mol. Cancer Ther. 13 (12), 3152–3162. 10.1158/1535-7163.MCT-14-0448 25349304

[B47] Manresa C. Sanz-Miralles E. C. Twigg J. Bravo M. (2018). Supportive periodontal therapy (SPT) for maintaining the dentition in adults treated for periodontitis. Cochrane Database Syst Rev 1 (1), CD009376. 29291254 10.1002/14651858.CD009376.pub2 PMC6491071

[B48] Martinez-Nieto G. Heljasvaara R. Heikkinen A. Kaski H. K. Devarajan R. Rinne O. (2021). Deletion of Col15a1 modulates the tumour extracellular matrix and leads to increased tumour growth in the MMTV-PyMT mouse mammary carcinoma model. Int. J. Mol. Sci. 22 (18), 9978. 10.3390/ijms22189978 34576139 PMC8467152

[B49] Michelson C. Al-Abedalla K. Wagner J. Swede H. Bernstein E. Ioannidou E. (2022). Lack of attention to sex and gender in periodontitis-related randomized clinical trials: a meta-research study. J. Clin. Periodontol. 49 (12), 1320–1333. 10.1111/jcpe.13707 35924761 PMC9669099

[B50] Molinelli E. Ceccarelli G. Fantone S. Di Mercurio E. Gambini D. Maurizi A. (2023). Melanoma and subcutaneous adipose tissue: role of peritumoral adipokines in disease characterization and prognosis. Pigment. Cell Melanoma Res. 36 (5), 423–430. 10.1111/pcmr.13103 37334675

[B51] Mutolo M. J. Morris K. J. Leir S. H. Caffrey T. C. Lewandowska M. A. Hollingsworth M. A. (2012). Tumor suppression by collagen XV is independent of the restin domain. Matrix Biol. 31 (5), 285–289. 10.1016/j.matbio.2012.03.003 22531369 PMC3373166

[B52] Nascimento G. G. Alves-Costa S. Romandini M. (2024). Burden of severe periodontitis and edentulism in 2021, with projections up to 2050: the Global Burden of Disease 2021 study. J. Periodontal Res. 59 (5), 823–867. 10.1111/jre.13337 39192495

[B53] Nguyen J. M. Jezequel P. Gillois P. Silva L. Ben Azzouz F. Lambert-Lacroix S. (2021). Random forest of perfect trees: concept, performance, applications and perspectives. Bioinformatics 37 (15), 2165–2174. 10.1093/bioinformatics/btab074 33523112 PMC8352507

[B54] Pischon N. Pischon T. Kroger J. Gulmez E. Kleber B. M. Bernimoulin J. P. (2008). Association among rheumatoid arthritis, oral hygiene, and periodontitis. J. Periodontol. 79 (6), 979–986. 10.1902/jop.2008.070501 18533773

[B55] Quadrianto N. Ghahramani Z. (2015). A very simple safe-bayesian random forest. IEEE Trans. Pattern Anal. Mach. Intell. 37 (6), 1297–1303. 10.1109/TPAMI.2014.2362751 26357350

[B56] Rasi K. Hurskainen M. Kallio M. Staven S. Sormunen R. Heape A. M. (2010). Lack of collagen XV impairs peripheral nerve maturation and, when combined with laminin-411 deficiency, leads to basement membrane abnormalities and sensorimotor dysfunction. J. Neurosci. 30 (43), 14490–14501. 10.1523/JNEUROSCI.2644-10.2010 20980607 PMC6634795

[B57] Rath-Deschner B. Memmert S. Damanaki A. Nokhbehsaim M. Eick S. Cirelli J. A. (2020). CXCL1, CCL2, and CCL5 modulation by microbial and biomechanical signals in periodontal cells and tissues-*in vitro* and *in vivo* studies. Clin. Oral Investig. 24 (10), 3661–3670. 10.1007/s00784-020-03244-1 32124070

[B58] Rhee S. Paik D. T. Yang J. Y. Nagelberg D. Williams I. Tian L. (2021). Endocardial/endothelial angiocrines regulate cardiomyocyte development and maturation and induce features of ventricular non-compaction. Eur. Heart J. 42 (41), 4264–4276. 10.1093/eurheartj/ehab298 34279605 PMC8560211

[B59] Ritchie M. E. Phipson B. Wu D. Hu Y. Law C. W. Shi W. (2015). Limma powers differential expression analyses for RNA-sequencing and microarray studies. Nucleic Acids Res. 43 (7), e47. 10.1093/nar/gkv007 25605792 PMC4402510

[B60] Schupp J. C. Manning E. P. Chioccioli M. Kamp J. C. Christian L. Ryu C. (2023). Alveolar vascular remodeling in nonspecific interstitial pneumonia: replacement of normal lung capillaries with COL15A1-positive endothelial cells. Am. J. Respir. Crit. Care Med. 208 (7), 819–822. 10.1164/rccm.202303-0544LE 37552025 PMC10563189

[B61] Shinjo T. Onizuka S. Zaitsu Y. Ishikado A. Park K. Li Q. (2023). Dysregulation of CXCL1 expression and neutrophil recruitment in insulin resistance and diabetes-related periodontitis in male mice. Diabetes 72 (7), 986–998. 10.2337/db22-1014 37058471 PMC10281234

[B62] Silva R. L. Lopes A. H. Guimaraes R. M. Cunha T. M. (2017). CXCL1/CXCR2 signaling in pathological pain: role in peripheral and central sensitization. Neurobiol. Dis. 105, 109–116. 10.1016/j.nbd.2017.06.001 28587921

[B63] Teles F. Collman R. G. Mominkhan D. Wang Y. (2022). Viruses, periodontitis, and comorbidities. Periodontol 89 (1), 190–206. 10.1111/prd.12435 35244970

[B64] Trost Z. Trebse R. Prezelj J. Komadina R. Logar D. B. Marc J. (2010). A microarray based identification of osteoporosis-related genes in primary culture of human osteoblasts. Bone 46 (1), 72–80. 10.1016/j.bone.2009.09.015 19781675

[B65] Wang H. Yang F. Luo Z. (2016). An experimental study of the intrinsic stability of random forest variable importance measures. BMC Bioinforma. 17, 60. 10.1186/s12859-016-0900-5 PMC4739337 26842629

[B66] Wang L. Zhang Y. L. Lin Q. Y. Liu Y. Guan X. M. Ma X. L. (2018). CXCL1-CXCR2 axis mediates angiotensin II-induced cardiac hypertrophy and remodelling through regulation of monocyte infiltration. Eur. Heart J. 39 (20), 1818–1831. 10.1093/eurheartj/ehy085 29514257

[B67] Wang S. Bai J. Zhang Y. L. Lin Q. Y. Han X. Qu W. K. (2022). CXCL1-CXCR2 signalling mediates hypertensive retinopathy by inducing macrophage infiltration. Redox Biol. 56, 102438. 10.1016/j.redox.2022.102438 35981418 PMC9418605

[B68] Wiggs J. L. Howell G. R. Linkroum K. Abdrabou W. Hodges E. Braine C. E. (2013). Variations in COL15A1 and COL18A1 influence age of onset of primary open angle glaucoma. Clin. Genet. 84 (2), 167–174. 10.1111/cge.12176 23621901 PMC3771394

[B69] Wu T. Hu E. Xu S. Chen M. Guo P. Dai Z. (2021). clusterProfiler 4.0: a universal enrichment tool for interpreting omics data. Innov. (Camb) 2 (3), 100141. 10.1016/j.xinn.2021.100141 PMC8454663 34557778

[B70] Yu S. Yi M. Xu L. Qin S. Li A. Wu K. (2019). CXCL1 as an unfavorable prognosis factor negatively regulated by DACH1 in non-small cell lung cancer. Front. Oncol. 9, 1515. 10.3389/fonc.2019.01515 31998654 PMC6966305

[B71] Zhan C. Zhou Z. Huang Y. Huang S. Lin Z. He F. (2023). Exploration of the shared gene signatures and molecular mechanisms between periodontitis and inflammatory bowel disease: evidence from transcriptome data. Gastroenterol. Rep. (Oxf) 11, goad041. 10.1093/gastro/goad041 37456714 PMC10348870

[B72] Zheng Y. Wang N. Wang S. Zhang J. Yang B. Wang Z. (2023). Chronic psychological stress promotes breast cancer pre-metastatic niche formation by mobilizing splenic MDSCs via TAM/CXCL1 signaling. J. Exp. Clin. Cancer Res. 42 (1), 129. 10.1186/s13046-023-02696-z 37210553 PMC10199643

